# Expression and existence forms of mast cell activating molecules and their antibodies in systemic lupus erythematosus

**DOI:** 10.1002/iid3.567

**Published:** 2021-11-16

**Authors:** Yuping Wang, Tengkai Wang, Meijuan Cai, Shuzhen Zhu, Lijun Song, Qian Wang

**Affiliations:** ^1^ Department of Laboratory Medicine Qilu Hospital (Qingdao), Cheeloo College of Medicine, Shandong University Qingdao Shandong China; ^2^ Department of Internal Medicine Qilu Hospital of Shandong University Jinan Shandong China; ^3^ Department of Rheumatology Qilu Hospital of Shandong University Jinan Shandong China; ^4^ Department of Laboratory Medicine Qilu Hospital of Shandong University Jinan Shandong China

**Keywords:** Anti‐FcεRI, Anti‐IgE, FcεRIα, IgE, SLE

## Abstract

**Introduction:**

Mast cells are regarded as a kind of classical anaphylaxis cells. However autoimmune diseases and allergic reactions have many similarities or overlaps. A large number of papers have proved that mast cells play a significant role in the pathogenesis of systemic lupus erythematosus (SLE). It is speculated that IgE, anti‐IgE antibodies, FcεRI, and anti‐FcεRI antibodies activate mast cells through autoimmune pathways and participate in the disease process of SLE. Naturally occurring protein molecules not only exist in monomer form, but also in polymer of protein molecules. Therefore, whether IgE, FcεRIα, anti‐IgE antibodies, and anti‐FcεRI antibodies also exist in polymeric forms in the natural state is worthy of further investigation.

**Methods:**

The serum samples and clinical data of 131 patients with SLE were collected from Qilu Hospital (Qingdao). Sixty healthy individuals were collected as the control group. Serum FcεRIα, anti‐IgE, and anti‐FcεRI were detected by enzyme‐linked immunosorbent assay. Serum IgE was detected by rate scatter nephelometry. A Chinese hamster ovarian cancer cell line CHO3D10 transfected with human FcεRIα was cultured and the cell protein extract was prepared. The existence forms of FcεRIα in the cell protein extract were detected by the native‐page method.

**Results:**

The serum FcεRIα in SLE patients was significantly higher than that in control group (3.52 [2.18, 4.71] µg/ml and 1.87 [1.52, 2.33] µg/ml, respectively; *p* < .05). Anti‐IgE was significantly lower than that in the control group (0.85 [0.55, 1.21] µg/ml and 1.23 [0.95, 1.58] µg/ml, respectively; *p* < .05). The CHO3D10 cell line expressed the FcεRIα, which had one kind of monomer (mFcεRIα) and two kinds of polymers (pFcεRIα) in the degeneration conditions.

**Conclusion:**

In patients with SLE, the expression of FcεRIα was increased and the level of anti‐IgE was decreased. FcεRIα had one kind of monomer and two kinds of polymers. Mast cell‐associated FcεRIα involved in the inflammatory lesion of SLE.

## Graphical abstract

First, as autoimmune diseases and allergic reactions have many similarities or overlaps, we compared the differences in serum IgE, FcεRIα, anti‐IgE, and anti‐FcεRI levels of systemic lupus erythematosus group and healthy control group for the first time. The serum FcεRIα in SLE patients is significantly higher than that in the control group and anti‐IgE is significantly lower than that in the control group, which provides a new reference basis for the understanding and further discussion of the inflammatory mechanism of the disease. Second, this study has revealed that the FcεRIα has one kind of monomer and two kinds of polymers in the degeneration conditions, which is worthy of further investigation.

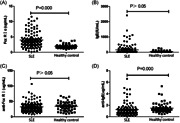

## 1 INTRODUCTION

Systemic lupus erythematosus (SLE) is an autoimmune disease involving multiple systems and organs and has multiple autoantibodies which are caused by the combined influence of genetics, hormones, and environmental factors. The pathogenesis is resulted from the breakdown of the homeostasis of apoptotic bodies, leading to the proliferation of self‐reactive T and B cells, and the production of autoantibodies against dsDNA, Sm, SS‐A, and SS‐B.^1^, ^2^

However, in recent years, researchers have paid more and more attention to other inflammatory cells except for T and B cells. For example, a large number of papers have proved that mast cells play a significant role in the pathogenesis of SLE.^3^ Mast cells and their activation‐related antibodies are involved in the occurrence and development of various autoimmune diseases such as rheumatoid arthritis and multiple sclerosis.^4^ The previous research also found that the levels of FcεRIα, anti‐IgE antibodies, and anti‐FcεRI antibodies related to the activation of mast cells in Graves' disease were higher than those in the healthy control group.^5^ It is speculated that IgE, anti‐IgE antibodies, FcεRI, and anti‐FcεRI antibodies activate mast cells through autoimmune pathways and participate in the disease process of SLE.

Naturally occurring protein molecules not only exist in monomer form, but also in polymer of protein molecules.^6^ Studies have reported that CRP not only exists in the form of monomer, but there are also multiple polymers of CRP in serum in the natural state, and perform different functions.^7^ Using the native‐page method to detect dog recombinant antigens Can f 1 and Can f 2 also showed the existence of dimer structure.^8^ Therefore, whether IgE, FcεRIα, anti‐IgE antibodies, and anti‐FcεRI antibodies also exist in polymeric forms in the natural state is worthy of further investigation.

Based on the above research foundation, this study systematically analyzed the expression levels of IgE, FcεRIα, anti‐IgE antibodies, and anti‐FcεRI antibodies in the serum of SLE patients, and explored their existence forms in the serum of SLE patients.

## 2 OBJECT AND METHODS

### 2.1 Object

#### 2.1.1 SLE patient group

Choosing 131 SLE patients treated between January and December 2019 in Qilu Hospital (Qingdao), Cheeloo College of Medicine, Shandong University, including 17 males and 114 females, aged 14.00–75.00 (38.50 ± 13.58) years old. These patients were all newly diagnosed according to the 1997 ACR revised criteria without allergic history. The autoantibody test results of SLE patients were different. The degree of abnormality and the degree of disease activity in SLE patients were scored (Table 1).

**Table 1:** Autoantibody levels in patients with SLE

	% (*n*)
Male/female	17/114
ANA	90.07 (118/131)
anti‐dsDNA	36.64 (48/131)
anti‐Sm	16.79 (22/131)
anti‐SSA	32.06 (42/131)
anti‐SSB	19.85 (26/131)
anti‐RNP	28.24 (37/131)
SLEDAI	11.00 (7.00, 14.00)

Abbreviations: ANA, anti‐nuclear antibody; anti‐dsDNA, anti‐double‐stranded DNA antibody; anti‐Sm, anti‐nucleoprotein antibody; anti‐SSA, anti‐SSA antibody; anti‐SSB, anti‐SSB antibody; anti‐RNP, anti‐ribonucleoprotein antibody; SLEDAI, SLE disease activity score.

#### 2.1.2 Healthy control group

Sixty cases of the healthy control group, including 20 males and 40 females, aged 31.00–57.00 (45.64 ± 6.47) years old, excluded SLE, allergies, and other autoimmune diseases, and autoantibody testing, liver and kidney function, and routine laboratory tests of hematuria were normal.

#### 2.1.3 CHO3D10 cells

CHO3D10 cell line is a Chinese hamster ovarian cancer cell line. Our research group transfected human FcεRIα gene clone into CHO3D10 cells through plasmid transfection, and obtained CHO3D10 cells with overexpression of human FcεRIα.

This study was approved by the ethics committee of Qilu Hospital (Qingdao), Cheeloo College of Medicine, Shandong University.

### 2.2 Methods

#### 2.2.1 Serum antinuclear antibody detection

Indirect immunofluorescence method (EUROIMMUN Antinuclear Antibody IgG Detection Kit).

#### 2.2.2 Serum anti‐dsDNA antibody detection

Indirect enzyme‐linked immunosorbent assay (ELISA) method (Shanghai Kexin Bio Anti‐double‐stranded DNA (dsDNA) Antibody Detection Kit).

#### 2.2.3 Serum IgE detection

Rate scatter turbidimetry (Siemens BNII instrument and its supporting reagents).

#### 2.2.4 Serum FcεRIα, anti‐IgE antibody, and anti‐FcεRI antibody detection

Competition ELISA method (BlueGene Biotech ELISA Kit).

#### 2.2.5 CHO3D10 cell protein extract

CHO3D10 cells transfected with human FcεRIα were resuscitated and cultured, washed twice with pre‐cooled phosphate‐buffered saline (PBS), when it was 80%–90% of the 9 cm diameter petri dish. Then we added 1 ml PBS to the petri dish and scraped the cells with the cell scraping, and collected the cell suspension in an EP tube. The whole process was operated on an icebox. The cell suspension was centrifuged at 1500g*10 min, and washed two times with pre‐cooled PBS, 2500g*5 min. Remove the supernatant, add the cell lysate according to the ratio of 10^6^ mouse‐derived cells corresponding to 20 µl lysate (1 mol/L Tris–Hcl pH = 7.5 3 ml, 10% SDS 10 ml, distilled water to 50 ml) to CHO3D10 cells. Metal bath 100°C, 10 min, until making the protein into fluid and transparent.

#### 2.2.6 CHO3D10 cell protein extract concentration detection

Refer to Biyuntian BCA Protein Concentration Determination Kit (enhanced).

#### 2.2.7 Native‐page method to detect the existence forms of FcεRⅠα expressed by CHO3D10 cells

CHO3D10 cell protein extract was subjected to native‐page electrophoresis and transferred to membrane, fixed in acetic acid fixative (8 ml of acetic acid, distilled water to 100 ml) for 15 min, and then the membrane was washed, closed. Mouse anti‐human FcεRIα (Ebioscience; 1:1000) was used as the primary antibody, and anti‐mouse IgG (Dako) conjugated with HRP (1:2000) was used as the secondary antibody. The reaction signal was chemiluminescent by ECL substrate (Amersham) detection.^9^, ^10^ Native‐page electrophoresis instrument and its supporting reagents (Life).

### 2.3 Statistical analysis

All statistical analyses were performed using SPSS 21.0 analysis software. Mast cell activation‐related antibodies and molecules IgE, FcεRIα, anti‐IgE, and anti‐FcεRI quantitative detection data showed a skewed distribution, represented by M (P25, P75), and the Mann–Whitney *U* rank‐sum test was used for comparison between groups. The difference was statistically significant with *p* < .05.

## 3 RESULTS

### 3.1 Results of serum IgE, FcεRIα, anti‐IgE antibody, and anti‐FcεRI antibody levels in SLE patient group and healthy control group

**Figure 1:**
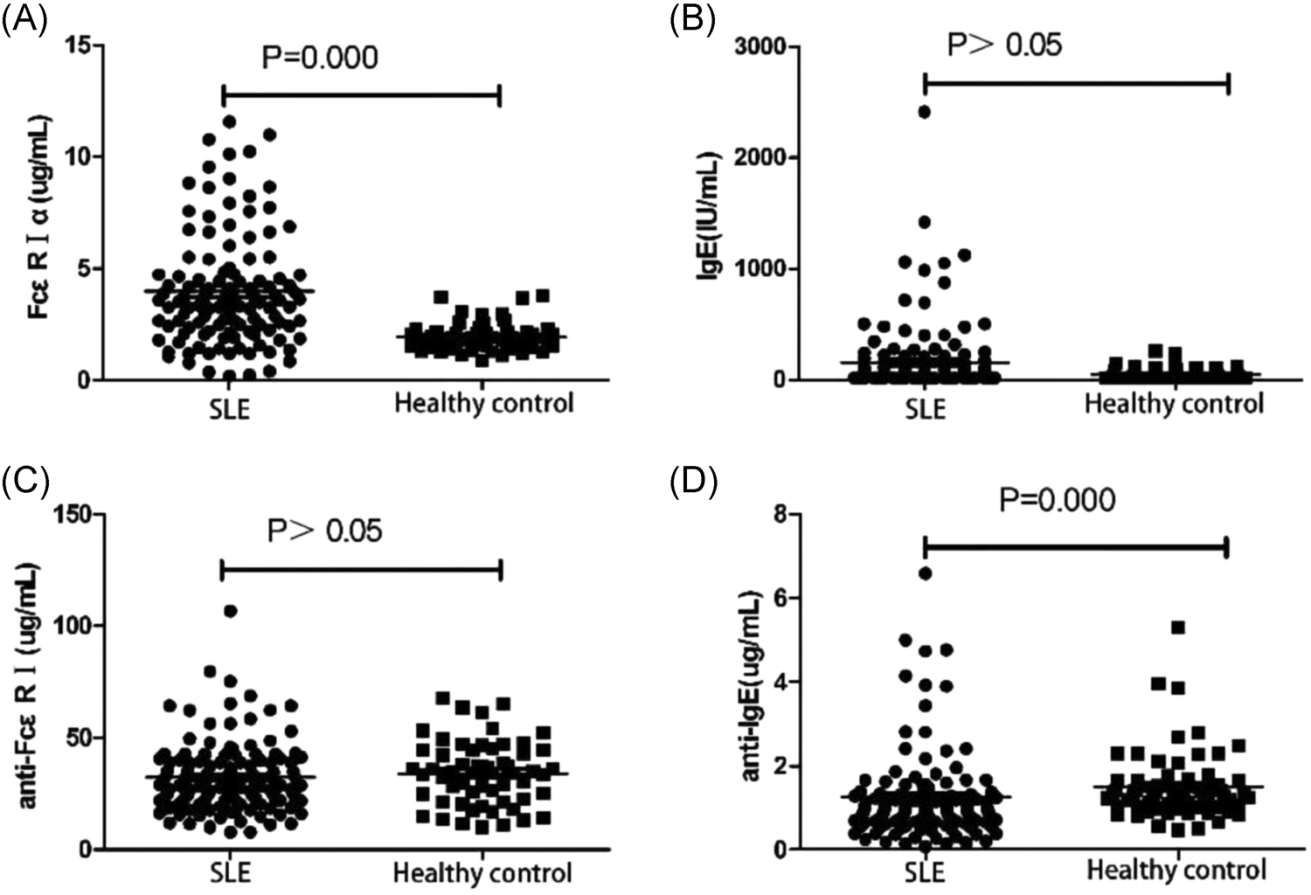
Comparison of serum IgE, FcεRIα, anti‐IgE antibody, and anti‐FcεRI antibody levels

There was no significant difference in the detection results of serum IgE and anti‐FcεRI antibodies between the SLE patient group and the healthy control group (*p* > .05). Serum FcεRIα: 3.52 (2.18, 4.71) µg/ml in SLE patient group was significantly higher than that in healthy control group: 1.87 (1.52, 2.33) µg/ml; serum anti‐IgE antibody level in SLE patient group: 0.85 (0.55, 1.21) µg/ml was significantly lower than that in healthy control group: 1.23 (0.95, 1.58) µg/ml (*p* = .000; Figure 1).

### 3.2 Morphological observation of CHO3D10 cells

**Figure 2:**
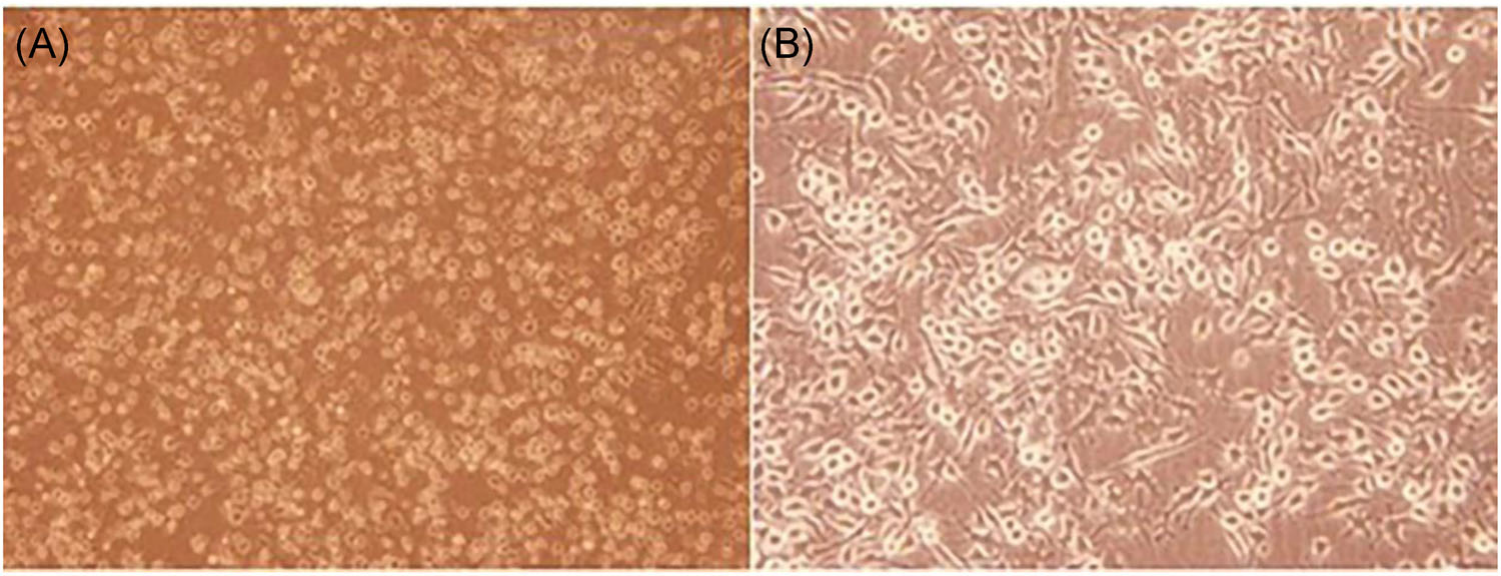
Morphology of CHO3D10 cells. (A) Amplification of microscope (100×); (B) amplification of microscope (200×) [Color figure can be viewed at wileyonlinelibrary.com]

Under the inverted microscope, it can be seen that the cells adhere to the wall, evenly distributed, the cell size was relatively uniform, fusiform or polygonal, and good refractive index (Figure 2).

### 3.3 Native‐page method to detect the existence forms of FcεRⅠα expressed by CHO3D10 cells

**Figure 3:**
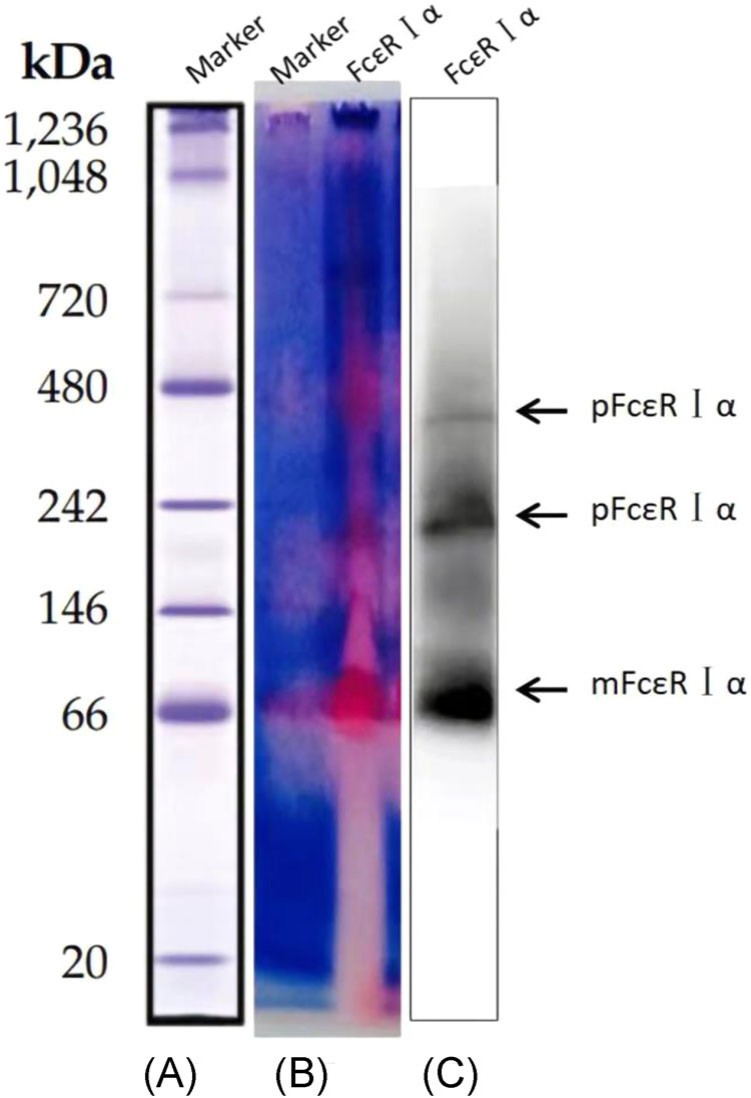
Native‐page showed the existence forms of FcεRⅠα. (A) Marker proteins: Refer to the “NativeMark™ Unstained Protein Standard Protocol.” (B) Marker and FcεRⅠα proteins were stained with Ponceau S Staining Solution. (C) FcεRⅠα proteins were transferred to polyvinylidene difluoride membranes for Western blot analysis. mFcεRⅠα, FcεRⅠα monomer; pFcεRⅠα, FcεRⅠα polymer [Color figure can be viewed at wileyonlinelibrary.com]

The protein extract concentration of the extracted CHO3D10 cells was determined by the BCA method to be 0.86 µg/µl. Native‐page method was used to detect the existence forms of FcεRⅠα. The results showed that FcεRⅠα existed in three forms under non‐denaturing conditions: one monomer (mFcεRⅠα) and two polymers (pFcεRⅠα) forms, and mFcεRⅠα had the most content (Figure 3).

## 4 DISCUSSION

Mast cells are regarded as a kind of classical anaphylaxis cells.^11^, ^12^ However autoimmune diseases and allergic reactions have many similarities or overlaps. Studies have also paid attention to the increase of mast cells in diseases such as Graves' ophthalmopathy, allergic rhinitis, SLE, and Sjogren's syndrome.^13^, ^14^ The results of this study showed that the level of FcεRIα in the serum of patients with SLE was significantly higher than that in healthy controls (*p* = .000), however, FcεRIα is the result of over‐transcription of DNA encoding FcεRIα during cell activation induced by FcεRI cross‐linking on the surface of mast cells.^15^ This study speculated that the cross‐linking FcεRI has activated the mast cells and contributed to the injury of chronic tissue damage during the pathogenesis of SLE.

Anti‐IgE antibodies and anti‐FcεRI antibodies are the results of the body's autoimmunity against excess IgE and FcεRIα, leading to complex and diverse forms of IgE, FcεRIα, anti‐IgE antibodies, and anti‐FcεRI antibodies. IgE and anti‐IgE antibodies, FcεRIα and anti FcεRI antibodies can form immune complexes.^16^, ^17^, ^18^ Miescher et al.^19^ reported that only when FcεRIα is not bound by IgE, anti‐FcεRI antibodies can bind to the cell surface FcεRIα, activate peripheral blood basophils, and cause inflammation. The results of this study also verified that there was no significant difference in serum IgE levels between the SLE group and the healthy control group. As a result, the autoimmunity of IgE and anti‐IgE antibodies caused the anti‐IgE antibody level in the SLE patient group to be significantly lower than that of the healthy control group, while the autoimmune result of FcεRIα and anti‐FcεRI antibody triggers tissue inflammatory damage caused by mast cell activation, which is worthy of further study and clarification.

As patients have different disease phenotypes and antibody detection results, I performed SLEDAI scores for SLE activity. The correlation between serum IgE, FcεRIα, anti‐IgE antibody, and anti‐FcεRI antibody levels in SLE patients and SLEDAI was analyzed. The results showed that there was no correlation between these four substances and SLEDAI, so this part was not included in the article.

As protein macromolecules, IgE, FcεRIα, anti‐IgE antibodies, and anti‐FcεRI antibodies must have a variety of complex forms in nature, such as BCR, CRP, recombinant antigens, and other biological macromolecules that all have monomers, dimers, and multiple polymers.^20^, ^21^ However, when we detected the existence forms of FcεRIα in serum, we did not get good detection results. The reason may be that IgE, anti‐IgE, and anti‐FcεRI antibodies in serum can form immune complexes with FcεRIα and affect the detection result. In this study, the CHO3D10 cells transfected with human FcεRIα was used as the research object, and the interference of IgE, anti‐IgE, FcεRⅠα, and anti‐FcεRI antibodies in the formation of immune complexes in the serum was excluded, and the native‐page method was used to detect FcεRⅠα protein molecule in its natural state. The results showed that the FcεRⅠα expressed by CHO3D10 cells existed in one monomer and two polymers under non‐denaturing conditions, but mFcεRⅠα had the most content. It implicates that although the protein macromolecules exist polymer under the natural state, monomer is still the main form of existence, which also explains why the deviation caused by the evaluation of traditional detection methods is still within the acceptable range. However, whether the existence of this polymer is a form of storage of protein molecules in the body, or whether it is a potential cause of disease in the body's environmental disorder still needs to be further explored.

In summary, IgE, FcεRIα, anti‐IgE antibodies, and anti‐FcεRI antibodies are the key factors in activating mast cells. An in‐depth study of the relationship between the existence of IgE, anti‐IgE, FcεRⅠα, anti‐FcεRI antibodies, and SLE provides a new reference basis for the understanding and further discussion of the inflammatory mechanism of the disease.

## CONFLICT OF INTERESTS

The authors declare that there are no conflict of interests.

## AUTHOR CONTRIBUTIONS

*Conception and design of the study*: Yuping Wang and Qian Wang. *Acquisition of data*: Tengkai Wang and Meijuan Cai. *Analysis and interpretation of data*: Shuzhen Zhu and Lijun Song. *Drafting the article*: Yuping Wang and Tengkai Wang. *Revising it critically for important intellectual content*: Qian Wang. *Final approval of the version to be submitted*: Yuping Wang, Tengkai Wang, Meijuan Cai, Shuzhen Zhu, Lijun Song, and Qian Wang.
